# Supplementary material for: Advanced Detection and Therapeutic Monitoring of Atherosclerotic Plaque Using CD36-Targeted Lipid Core Probe
Source: Pharmaceutics. 2025 Mar 30;17(4):444. doi: 10.3390/pharmaceutics17040444 (PMC12030024; doi:10.3390/pharmaceutics17040444)
Supplement: Supplementary file 1 [file pharmaceutics-17-00444-s001.zip › pharmaceutics-3449582-supplementary.pdf]

Advanced Detection and Therapeutic Monitoring of Atherosclerotic Plaque Using CD36-Targeted Lipid Core Probe

Tingting Gao[1†], Siqi Gao[1†], Maolin Qiao[1], Chuanlong Lu[1], Heng Wang[1,2], Hongjiu Zhang[1], Lizheng Li[1], Shule Wang[1], Ruijing Zhang[1*] and Honglin Dong[1*]

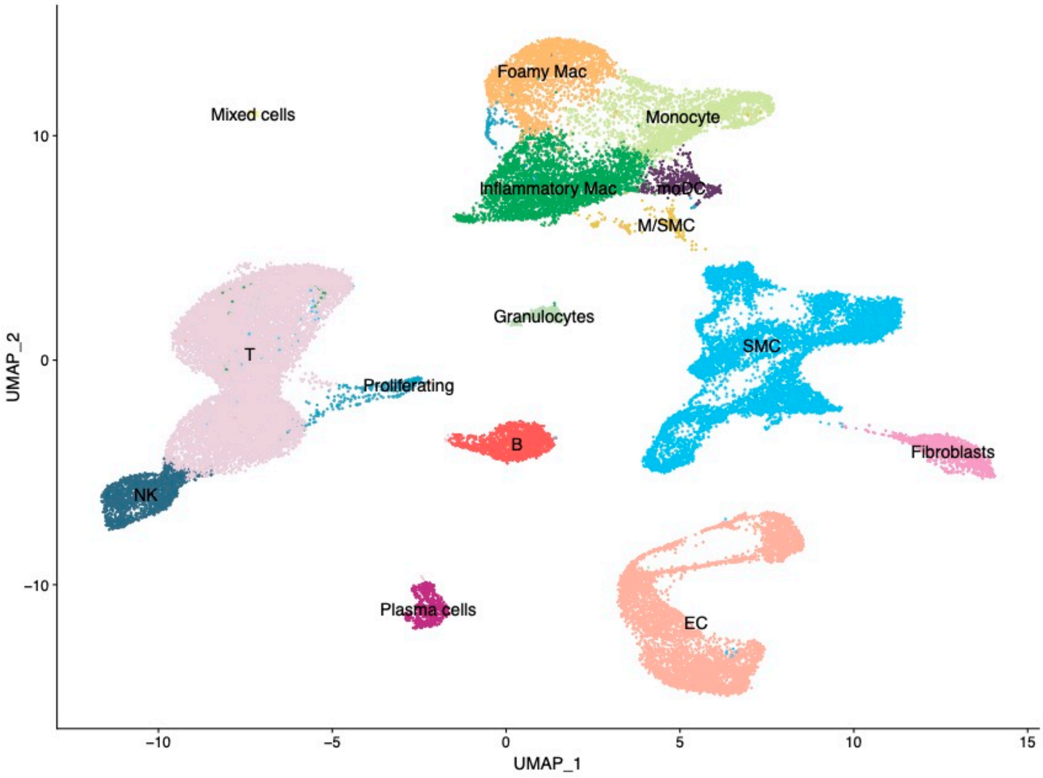

Supplement Figure S1. Visualization of 15 clusters of carotid plaque in UMAP.

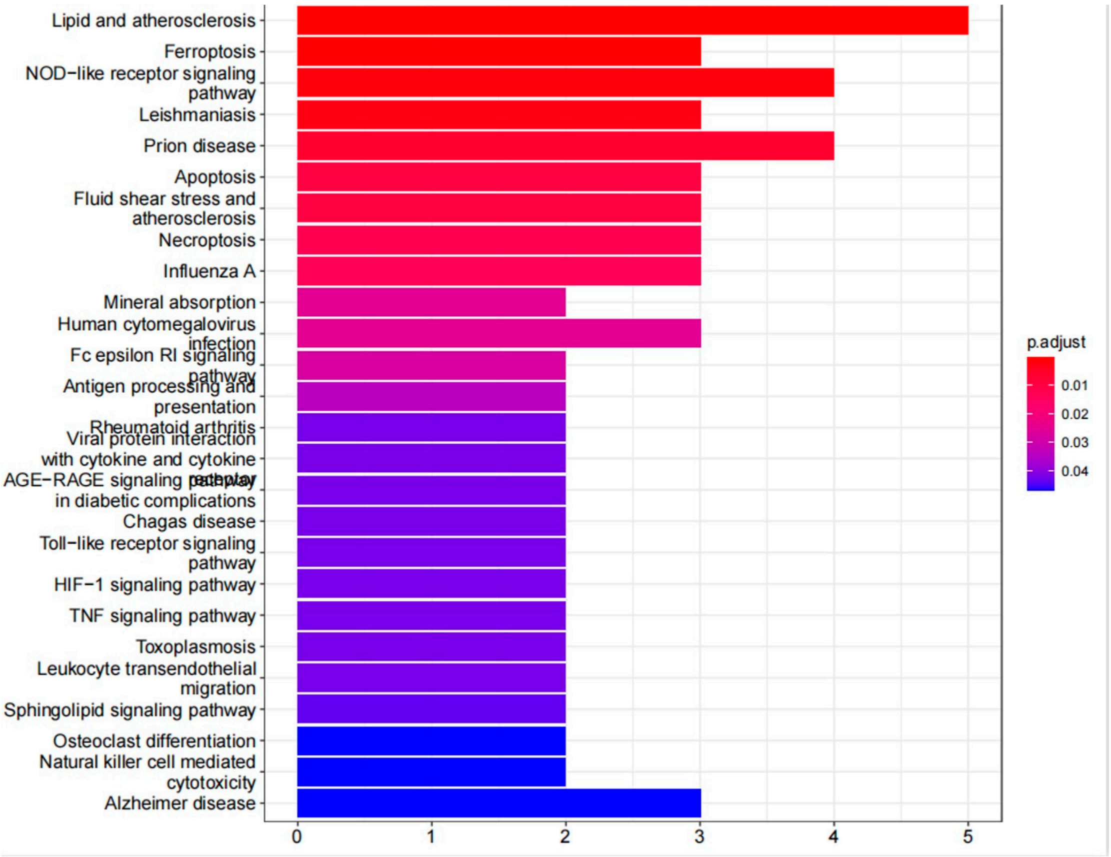

Supplement Figure S2. KEGG analysis showed the key signal pathway of AS.

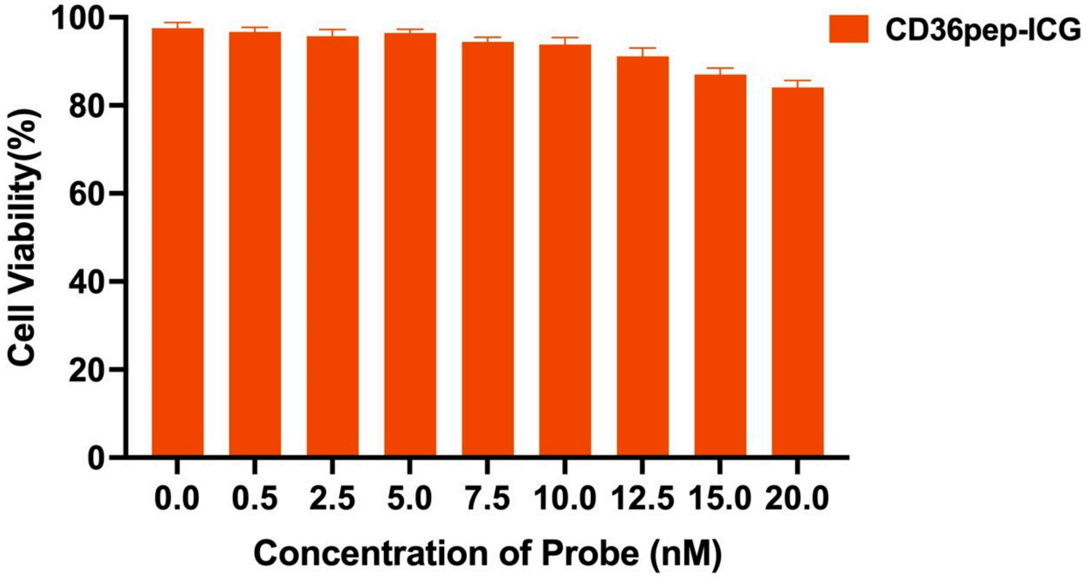

Supplement Figure S3. CCK-8 assay of Raw264.7 cells demonstrated no obvious cytotoxicity after 24 h of incubation with diverse concentrations of the Probe.
